# Endoscopic Treatment for Pediatric Esophageal Stenosis Induced by Chemical Burn, Congenitally, or After Surgical Repair of Esophageal Atresia

**DOI:** 10.3389/fped.2022.814901

**Published:** 2022-02-25

**Authors:** Bingyi Zhou, Hailing Peng, Liu Han, Chengbai Liang, Liang Lv, Xuehong Wang, Deliang Liu, Yuyong Tan

**Affiliations:** ^1^Department of Gastroenterology, The Second Xiangya Hospital, Central South University, Changsha, China; ^2^Research Center of Digestive Disease, Central South University, Changsha, China

**Keywords:** esophageal stenosis, endoscopic treatment, esophageal atresia, endoscopic balloon dilation, endoscopic stent replacement, endoscopic incision

## Abstract

**Objectives:**

To evaluate the safety and efficacy of endoscopic treatment for congenital pediatric esophageal stenosis or pediatric stenosis that develops after a chemical burn or surgical repair of esophageal atresia.

**Methods:**

We retrospectively reviewed the medical records of 15 pediatric patients who underwent endoscopic treatments (dilation and/or stenting and/or incision) for congenital esophageal stenosis or esophageal stenosis that developed after a chemical burn or surgical repair of esophageal atresia, between January 2010 and January 2019. The patients were periodically followed-up to assess the safety and efficacy of treatment by comparing the diameter of stricture and dysphagia score before and after procedures, and complications or recurrence.

**Results:**

All children successfully underwent the procedures. Fourteen of the 15 patients received endoscopic balloon dilation (EBD) as the first step of treatment, but EBD alone only resolved the symptoms in two patients. The remaining patients received other comprehensive treatments, such as EBD with endoscopic incision (EI), EBD with stent replacement, or a combination of EBD, stent replacement, and EI. Eleven (11/15, 73.3%) patients experienced symptomatic relief after endoscopic treatment, and recurrence was noted in four patients on 3–36 months after the final endoscopic treatment. All four patients underwent esophageal surgery to relieve their symptoms. Until October 2021, all patients experienced symptom relief, and their dysphagia scores decreased from 3–4 to 0–1 during the follow-up period of 8–121 months. The average diameter of stenosis was increased from 0.34 cm (range 0.2–0.7 cm) to 1.03 cm (range 0.8–1.2 cm). No severe complications occurred during endoscopic treatment and follow-up.

**Conclusions:**

Endoscopic treatment is safe and effective for pediatric esophageal stenosis that is congenital or induced by chemical burns or surgical repair of esophageal atresia. Comparative large-scale studies are required to confirm our findings.

## Introduction

There are many causes of esophageal stenosis in children, including esophageal atresia, inflammatory diseases, gastroesophageal reflux disease, eosinophilic esophagitis, congenital esophageal stenosis (CES), surgical complications of esophageal atresia, and chemical burns (1–3). The most common etiologies vary among countries. Burns are common in developing countries (4, 5). Dysphagia and vomiting are common clinical manifestations in children with esophageal stenosis. These patients usually have long-term morbidity, and multiple procedures may be required to relieve their symptoms. Esophageal dilatation usually serves as the first-line treatment because it effectively relieves symptoms. However, stenosis recurrence is common and research has shown that it can be difficult to maintain a satisfactory lumen diameter for over 4 weeks (1). Currently, there is no established treatment for patients with refractory stenosis. Some researchers have reported successful application of esophageal stents, endoscopic incision (EI), or mitomycin C following dilation (6). However, the efficacy of these newly proposed modalities remains unclear. Therefore, we reviewed the medical records of pediatric patients with congenital esophageal stenosis or esophageal stenosis that developed secondary to chemical burns or surgical repair of esophageal atresia in our hospital, who were treated with different endoscopic procedures, including endoscopic balloon dilation (EBD), endoscopic stent placement, and endoscopic incision (EI), to investigate the treatment outcomes of these interventions.

### Patient Information

This retrospective study enrolled pediatric patients with congenital esophageal stenosis or stenosis that developed following a chemical burn or surgical procedure (surgical repair of esophageal atresia), who underwent endoscopic treatment in our hospital between January 2010 and January 2019. The study was approved by the Ethics Committee of the Second Xiangya Hospital of Central South University.

Data, including those relating to demographic, clinical characteristics, outcomes of endoscopic treatment, and follow-up, were extracted from the patients' medical records. The patients' guardians were informed about the possible treatment options (including surgical and endoscopic methods) and potential adverse events associated with each option, and informed consent was obtained from them. Pediatric patients with blood coagulation disorders or severe cardiopulmonary diseases were excluded from the study. Usually, EBD was recommended as the primary treatment for stenosis shorter than 5 cm, and ESP was suggested for stricture longer than 5 cm, for recurrent stenosis, repeated EBD, EI, ESP, or combined therapy of them may be suggested. Surgery was also a choice for recurrent stenosis. The final treatment method each time was achieved after consideration of various factors (such as guardians' will, our condition, doctor's experience, etc.).

All endoscopic treatments were performed under general anesthesia *via* tracheal intubation or under conscious sedation with the patient in the left lateral position. A single-channel endoscope (GIF-Q240/Q260J, Olympus) was used.

### EBD Procedure

EBD was performed using a polyethylene balloon system (Rigiflex, Microvasive, Boston Scientific). The balloon diameter, usually 1.0 or 1.5 cm, was selected based on the age of the patient and the diameter of the stenosis. We usually set the balloon to an initial pressure of 3 atm, and this could be gradually increased to 5–8 atm after endoscopic evaluation. Expansions were maintained for 1–2 min, with a 2–5 min interval between expansions. Endoscopic examination was performed during the interval to detect any serious complications and confirm that the stenosis was sufficiently loose. Dilatations were continued until confirmation of either a loosened stricture or complications, such as mucosal laceration or hemorrhage.

### Endoscopic Stent Placement Procedure

Esophageal stents were provided by Delman Technology Co. Ltd. (Jinan, Shandong, China). The length of the stent was selected based on the stricture length determined *via* endoscopy or imaging before the operation. Fully covered self-expandable metal stents are preferred because they can provide continuous dilation to the stenosis and are easy to remove after the relief of esophageal stenosis. Details of the steps involved in stent placement have previously been reported (7). After stent placement, endoscopy was repeated to confirm the appropriateness of the distance from the margin of the stent to the incisor.

### EI Procedure

**Figure 1:**
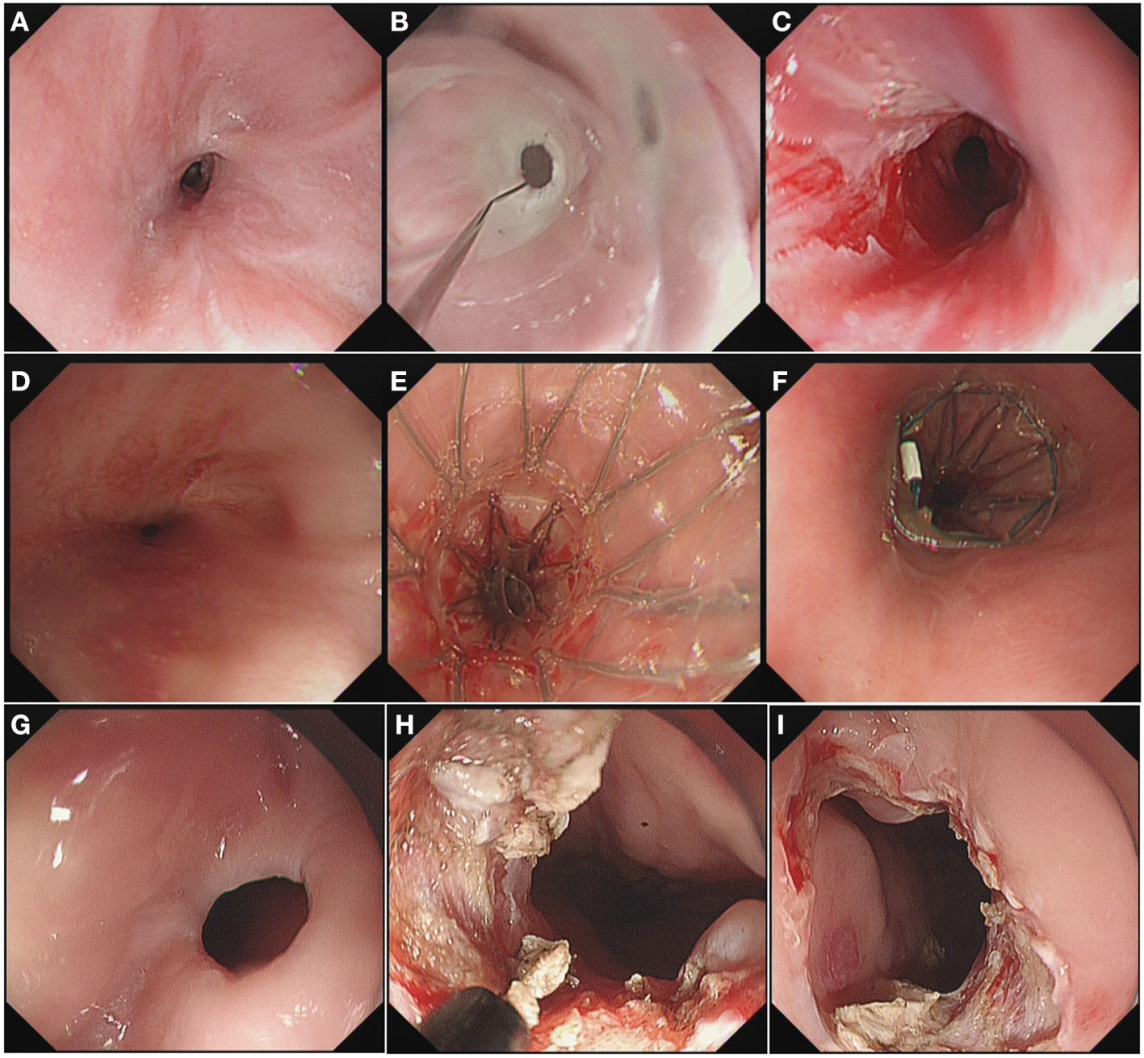
**(A)** Endoscopy Image of the stenosis before EBD. **(B)** EBD procedure. **(C)** Enlarged diameter of the stenosis after EBD. **(D)** Endoscopy Image of the stenosis before endoscopic stent placement. **(E)** Insertion of the stent. **(F)** Observation of the upper margin of the stent. **(G)** Endoscopy Image of the stenosis before EI. **(H)** Radial incisions under endoscopy. **(I)** Enlarged diameter of the stenosis after EI.

The steps used for EI were the same as those we previously reported (8). Under endoscopy, radial incisions were made along a virtual line connecting the oral end of the esophageal lumen and the anal end of the stenosis. Involvement of the muscularis propria and/or exposure of the bottom of the incision along the virtual line was regarded as a sufficient incision depth. After incision, the wound surface at the stenosis was carefully checked to confirm that there are no hemorrhages or perforations. Figure 1 depicts an example of EBD, endoscopic stent placement, and EI.

### Postoperative Management

All patients were kept *nil per os* for 1 day, then on a liquid diet for 2–3days, and gradually allowed a soft diet within a month. Prophylactic intravenous proton pump inhibitors (PPIs) and antibiotics were occasionally used. Patients who underwent EBD and/or EI were scheduled for endoscopic examination every 2–3 months for the first 6 months after treatment. For patients who underwent stent placement, monthly chest X-ray examinations were scheduled to check for stent migration before final stent removal, and endoscopy was performed as necessary to adjust, reset, or remove the stent. Endoscopy was performed on all the patients for as long as recurrent dysphagia was experienced.

The time for stent removal was determined if any of the following criteria were met: (1) the stent had been in place for as long as 12 weeks, even if there was no migration, (2) the stent had fallen into the stomach, but dysphagia was relieved, or, (3) severe tissue overgrowth or ulceration on the upper and/or lower edges of the stent was observed. The stent was replaced if it shifted from the original position without improvement in the stenosis.

### Evaluation of Dysphagia Before and After EI

The grade of dysphagia symptoms was defined by the following scoring system (9): 0, able to eat a normal diet; (1) unable to swallow certain solids, (2) able to swallow semisolid foods, (3) able to swallow liquids only, and 4, unable to swallow liquids.

### Definition of Failure and Indication of Repeat Treatment

Treatment was considered unsuccessful when an endoscope with a diameter of 5.9 mm could not be passed through and the patient's dysphagia score exceeded 2. Additional endoscopic or surgical treatment was scheduled after such treatment failures.

## Results

### Patient Characteristics

The 15 patients were aged 1–14 years old, of these, 13 were boys and 2 were girls. There were 18 strictures in the 15 patients. All the patients received at least one type of treatment. All preoperative dysphagia scores were either 3 or 4. Detailed clinical data are presented in Table 1.

**Table 1:** Treatment for esophageal stenosis after chemical burn.

**Patient no**.	**Sex/age, y**	**Major symptom**	**Therapy**	**Distance from the incisors, cm**	**Length of the stricture, cm**	**Diameter of stricture,cm**		**Dysphagia score**		**Stenting insertion**	**Complication/recurrence**	Follow-up^*^, mo
						**Pre**	**Post**	**Pre**	**Post**			
1	M/4	Dysphagia	1EBD 1EBD+Stenting	12 16	0.8 10	0.5 0.3	1.0 1.0	3	1	Yes 13 × 130	Subcutaneous emphysema	90
2	M/8	Dysphagia	1EBD 1EBD+Stenting	23	5	0.4	1.2	3	0	Yes 14 × 105	None	24
3	M/11	Dysphagia	1 EBD	14	3.0	0.2	1.2	4	1	No	None	72
4	M/14	Dysphagia	3 EBD 1EBD+Stenting	32	1.0	0.5	1.2	3	1	No	Recurrent 31 month later	36
5	M/5	Vomiting	8 EBD 1EBD+ Stenting 2EBD+EI	10 20	12 4	0.5 0.3	1.2 1	3	0	No	None	35
6	M/14	Vomiting	2 EBD	36	1.0	0.4	1.0	3	1	No	None	13
7	M/2	Dysphagia	3 EBD 2EBD+ Stenting	14	11	0.3	1	4	1	Yes 13 × 105	None	99
8	F/6	Vomiting	2EBD 2EBD+Stenting	16	3	0.2	1	4	1	Yes 14 × 85	Recurrent 9 month later	121

* *Refers to the follow-up duration after final treatment in our department*.

### Treatment Outcome and Adverse Events

A total of 80 sessions of endoscopic treatment were administered to the 15 patients. This included 42 EBD sessions, 7 EI sessions, 1 stenting session, 19 EBD+Stenting sessions, 5 EBD+EI sessions, and 6 EI+Stenting sessions. All three types of endoscopic treatment were performed successfully. Fourteen of the 15 patients received EBD as the first step for treatment, but only two patients had their symptoms relieved by EBD alone. For patient 12, stent replacement alone was sufficient to relieve his symptoms. The remaining 12 patients received comprehensive treatment, such as EBD with EI, EBD with stent replacement, or a combination of EBD, stent replacement, and EI. Excluding patient 15, the overall median (IQR) treatment intervals of EBD, EI, EBD+Stenting, EBD+EI, and EI+Stenting were 2(1, 3), 4(2.5, 6), 10(3.5, 32.5), 3(3, 2), and 3(3, 5.35) months, respectively. Sustained symptom improvement was achieved in 73.3% (11/15) of the patients during a follow-up period of 8–121 months after treatment. The diameter of stricture was enlarged from 0.34 cm (range 0.2–0.7 cm) to 1.03 cm (range 0.8–1.2 cm) on average. Patient 1 developed subcutaneous emphysema, which resolved within a week without analgesic therapy. Patients 4, 8, 13, and 14 experienced recurrences at 31, 9, 3, and 36 months, respectively, after the final endoscopic treatment, and all of these patients underwent esophageal surgery to relieve their symptoms (Tables 1–3).

**Table 2:** Treatment for esophageal stenosis after surgical repair of esophageal arteria.

**Patient no**.	**Sex/age, y**	**Major symptom**	**Therapy**	**Distance from the incisors, cm**	**Length of the stricture, cm**	**Diameter of stricture,cm**		**Dysphagia score**		**Stenting insertion**	**Complication/ recurrence**	Follow-up^*^, mo
						**Pre**	**Post**	**Pre**	**Post**			
9	F/5	Dysphagia	14EBD 2 EBD+Stenting	25	4	0.3	1.0	4	1	Yes 14 × 85	None	12
10	M/1	Vomiting	4EBD+Stenting	14	3.0	0.2	0.9	4	1	Yes 13 × 105	None	24
11	M/3	Dysphagia	2EBD 1EBD+Stenting	23	3	0.3	0.8	4	1	Yes 12 × 85	None	111
12	M/6	Dysphagia	1Stenting	24	4.5	0.7	1.0	3	0	Yes 14 × 85	None	121

* *Refers to the follow-up duration after final treatment in our department*.

**Table 3:** Treatment for congenital esophageal stenosis.

**Patient no**.	**Sex/age, y**	**Major symptom**	**Therapy**	**Distance from the incisors, cm**	**Length of the stricture, cm**	**Diameter of stricture,cm**		**Dysphagia score**		**Stenting insertion**	**Complication/recurrence**	Follow-up^*^, mo
						**Pre**	**Post**	**Pre**	**Post**			
13	M/3	Vomiting	3EBD 1 EI	16	9	0.2	1.0	4	1	No	Recurrent 3 month later	12
14	M/8	Vomiting	2 EBD+Stenting	18	2	0.4	1	3	1	Yes 14 × 105	Recurrent 36 month later	72
15	M/4	Vomiting	2 EBD 6 EI 2EBD+ Stenting 3EBD+EI 6EI+Stenting	16 22	2 2	0.2 0.2	1 1	4	1	Yes 14 × 120	None	8

* *Refers to the follow-up duration after final treatment in our department*.

## Discussion

The present study describes our experiences with endoscopic treatment of pediatric patients with esophageal stenosis that was congenital or induced by chemical burns or surgical repair of esophageal atresia. Unlike adult patients, in whom anastomotic stenosis after surgical resection of esophageal tumors is the leading source of stenosis, the etiological spectrum for children is comparatively broad. Nonetheless, the clinical presentations of children with esophageal stenosis tend to be very similar and are usually characterized by dysphagia, recurrent vomiting, and food impaction. Esophageal perforation may occur in some cases of corrosive ingestion (10). In addition to medical management, almost all patients with esophageal stenosis require either endoscopic dilatation or surgical intervention (11). To our knowledge, few articles discuss comprehensive endoscopic treatment of esophageal stenosis in children, as its incidence is relatively low worldwide.

Many reports have suggested that all endoscopic interventions should be performed under general anesthesia in pediatric patients (12). For children with esophageal stenosis, there have been many studies supporting EBD as a safe procedure with minimal morbidity and mortality, especially for anastomotic strictures, and those shorter than 5 cm (13, 14), making it a common first-line therapy for esophageal stenosis. Moreover, its safety and effectiveness have been emphasized, along with its long-term clinical success and nutritional promotion (15, 16). The size of the balloon catheter can vary from 0.4 to 2.2 cm, and the duration of balloon inflation can vary from 20 to 120 s (17). There is still no standard for the optimal timing of esophageal dilation. Two retrospective studies compared routine esophageal dilations (every 3 weeks, starting 3 weeks post-surgery) with dilations when symptoms developed in children post-esophageal atresia. They found no difference in outcomes or complications, but dilation times were significantly lower in the on-demand dilation group (18). Although more practical evidence is needed, the following rule of three has been widely accepted: dilate up to three times the diameter of stenosis, with an average of three dilations, and a minimum period of 3 weeks between dilation sessions (19).

Multiple EBDs are associated with a higher frequency of invasive operations and a greater risk of operation-related complications. Thus, endoscopic stent placement is prioritized. It has been widely accepted that temporary placement of self-expandable stents could be considered for refractory benign esophageal strictures in adult patients (20). For pediatric patients, Baghdadi et al. (21) performed a retrospective review of pediatric patients with esophageal atresia complicated by esophageal stenosis, in which patients who underwent endoscopic stent placement showed good outcomes after long-term follow-up. According to our prior experience, endoscopic stent placement is a good recommendation for pediatric patients with refractory esophageal stenosis, as it could provide continuous, radially oriented dilation pressures over a period of time. This is helpful for scar remodeling and decreasing the risk of perforation and restenosis (8). Moreover, for patients who undergo EBD and/or EI, damage to the esophageal wall is inevitable, and fully covered stents can protect the wound surface from gastric acid, thus potentially reducing the incidence of stenosis recurrence. Stents may remain for more than 4 weeks, which efficaciously lowers the frequency of invasive endoscopic operations and reduces the risk of complications during procedures. In this study, the median treatment interval was significantly longer in the EBD+stenting group than in the EBD group (10 vs. 4 months), which also confirmed the importance of stent placement after EBD. However, the migration rate after esophageal stent placement is high (22), and the possible complications including chest pain, reflux esophagitis, stent shedding, granulation tissue hyperplasia should not be ignored (23).

The efficacy and safety of EI have been proven in adults with anastomotic strictures and refractory benign strictures in recent years (24–31), but reports of its effectiveness in pediatric patients are rare. For adult patients with a Schatzki's ring, DiSario et al. (32) found that up to 64% of patients may relapse during follow-up, and the remission period was significantly longer with EI treatment than with EBD. The results of a prospective clinical trial involving 50 patients with Schatzki's rings showed that the asymptomatic survival of the EI group was higher than that of the EBD group (33). In our study, the median treatment interval of EI (4 months) was longer than that of EBD (2 months), which indicated that EI may result in longer asymptomatic survival than EBD. Additionally, EI could be an effective co-treatment for EBD and endoscopic stent placement. Our group (34) reported the case of a pediatric patient successfully treated with EI and esophageal stenting and performed an analysis of a case series (8) in which the symptoms of pediatric patients were successfully relieved after EI/EI+stent replacement when the previous attempts at dilation/stenting failed. It was found that EI efficacy was related to the stenosis length. Hordijk et al. (24) reported that patients with a stricture of <1.0 cm did not exhibit dysphagia recurrence during the 12 months of follow-up, whereas patients with a long-segment stricture size 1.5–5.0 cm required an average of three sessions to prevent a recurrence.

In a study by Muto et al. (25), a median of four sessions of preventive dilation were performed repeatedly to maintain patency. In our study, three patients underwent EI. Patient 13, who did not receive a stent replacement, suffered from recurrent dysphagia, supporting the notion that stent placement is beneficial for patients with a long-segment stricture. However, for patients who underwent EBD+EI or EI+Stenting, the treatment interval was 3(3, 2) months and 3(3, 5.35) months, respectively, which is shorter than that for EI alone 4(2.5, 6) months. However, for the EI+ stenting group, the P75 was significantly higher, suggesting that asymptomatic survival may be longer. However, as we only assessed five and six patients, respectively, in the EBD+EI and EI+Stenting groups, our findings need to be validated in a larger sample. Moreover, we suggest that EI should be performed by an experienced endoscopist, considering the risk of perforation due to the lack of epithelialization of the stricture.

In summary, EBD is suggested as the first and regular treatment step for children with esophageal stenosis shorter than 5 cm (22). Endoscopic stent placement is superior in maintaining continuous, radially oriented dilation pressures, and can serve as the first-line treatment for stenosis longer than 5 cm or recurrent stenosis. Furthermore, for patients who underwent EBD or EI, fully covered stents can protect the wound surface from gastric acid. EI could bring longer asymptomatic survival than EBD in our experience, and a combination therapy such as EBD+ESP, EI+ESP may result in a better efficacy for recurrent or refractory stenosis.

Moreover, there are other treatments for esophageal stenosis which we have not used. For example, an intralesional steroid injection may reduce the risk of recurrent stenosis by inhibiting collagen formation, accelerating collagen breakdown, and decreasing fibrotic healing (6). However, there is still a lack of consensus regarding the details of procedures involving optimal injection technique, frequency of injection, and dosage, especially for pediatric patients (35–37). Another example is mitomycin-C. Although studies have suggested that it may benefit patients with refractory strictures by decreasing collagen synthesis and scar formation (38–42), in a study conducted by Chapuy et al. (43), adding mitomycin-C treatment did not result in any benefit in strictures compared with repeated esophagea dilations alone. Thus, we did not choose to utilize these treatments for pediatric patients.

Our study has several limitations: first, this was a retrospective study with a relatively small sample size, therefore, selection and referral biases may exist. Second, this was a single-center study conducted in a tertiary hospital, and no patient received endoscopic drug therapy, such as intra-lesional injection of corticosteroids or mitomycin-C. Third, there was no comparison between endoscopic and surgical treatments, thus, it is difficult to confirm the advantages of endoscopic modalities. Furthermore, the comparison between different endoscopic treatments is limited because of the small number of patients, for example, only 3 patients received pure stent placement. Responses to different modalities of treatment for esophageal stenosis may also be different for esophageal atresia, chemical burns, and other causes of esophageal stenosis. Thus, larger comparative studies are required to confirm our findings and to suggest the best modality for pediatric esophageal stenosis.

In conclusion, our study showed that endoscopic treatment may serve as a safe and effective method for the treatment of pediatric esophageal stenosis—congenital or that caused by chemical burns or surgical repair of esophageal atresia. In most patients, endoscopic management (EBD, EI, stent placement, or a combination of these three methods) can relieve symptoms. Surgery is an alternative treatment option in the case of endoscopic failure.

## Data Availability Statement

The original contributions presented in the study are included in the article/supplementary material, further inquiries can be directed to the corresponding author/s.

## Ethics Statement

The studies involving human participants were reviewed and approved by the Ethics Committee of the Second Xiangya Hospital of Central South University. Written informed consent to participate in this study was provided by the participants' legal guardian/next of kin. Written informed consent was obtained from the minor(s)' legal guardian/next of kin for the publication of any potentially identifiable images or data included in this article.

## Author Contributions

BZ contributed to drafting the manuscript. HP and LH contributed to data analysis and picture collecting. CL, LL, and XW contributed to the descriptions of detailed operation steps. YT and DL contributed to the conception of the study and revising it critically for important intellectual content. All authors contributed to the article and approved the submitted version.

## Conflict of Interest

The authors declare that the research was conducted in the absence of any commercial or financial relationships that could be construed as a potential conflict of interest.

## Publisher's Note

All claims expressed in this article are solely those of the authors and do not necessarily represent those of their affiliated organizations, or those of the publisher, the editors and the reviewers. Any product that may be evaluated in this article, or claim that may be made by its manufacturer, is not guaranteed or endorsed by the publisher.
